# Validation of different automated segmentation models for target volume contouring in postoperative radiotherapy for breast cancer and regional nodal irradiation

**DOI:** 10.1016/j.ctro.2024.100855

**Published:** 2024-09-11

**Authors:** Eva Meixner, Benjamin Glogauer, Sebastian Klüter, Friedrich Wagner, David Neugebauer, Line Hoeltgen, Lisa A. Dinges, Semi Harrabi, Jakob Liermann, Maria Vinsensia, Fabian Weykamp, Philipp Hoegen-Saßmannshausen, Jürgen Debus, Juliane Hörner-Rieber

**Affiliations:** a Department of Radiation Oncology, Heidelberg University Hospital, Im Neuenheimer Feld 400, 69120 Heidelberg, Germany; b Heidelberg Institute of Radiation Oncology (HIRO), 69120 Heidelberg, Germany; c National Center for Tumor Diseases (NCT), Heidelberg, Germany; d Heidelberg Ion Therapy Center (HIT), Im Neuenheimer Feld 450, 69120 Heidelberg, Germany; e German Cancer Research Center (DKFZ), Clinical Cooperation Unit Radiation Oncology, Im Neuenheimer Feld 280, 69120 Heidelberg, Germany; f Department of Radiation Oncology, University Hospital Düsseldorf, Düsseldorf, Germany

**Keywords:** Deep learning segmentation, Auto-segmentation, AI contouring, Target volume delineation, Clinical implementation, Quality assurance, Machine learning, Artificial intelligence

## Abstract

**Introduction:**

Target volume delineation is routinely performed in postoperative radiotherapy (RT) for breast cancer patients, but it is a time-consuming process. The aim of the present study was to validate the quality, clinical usability and institutional-specific implementation of different auto-segmentation tools into clinical routine.

**Methods:**

Three different commercially available, artificial intelligence-, ESTRO-guideline-based segmentation models (M1-3) were applied to fifty consecutive reference patients who received postoperative local RT including regional nodal irradiation for breast cancer for the delineation of clinical target volumes: the residual breast, implant or chestwall, axilla levels 1 and 2, the infra- and supraclavicular regions, the interpectoral and internal mammary nodes. Objective evaluation metrics of the created structures were conducted with the Dice similarity index (DICE) and the Hausdorff distance, and a manual evaluation of usability.

**Results:**

The resulting geometries of the segmentation models were compared to the reference volumes for each patient and required no or only minor corrections in 72 % (M1), 64 % (M2) and 78 % (M3) of the cases. The median DICE and Hausdorff values for the resulting planning target volumes were 0.87–0.88 and 2.96–3.55, respectively. Clinical usability was significantly correlated with the DICE index, with calculated cut-off values used to define no or minor adjustments of 0.82–0.86. Right or left sided target and breathing method (deep inspiration breath hold vs. free breathing) did not impact the quality of the resulting structures.

**Conclusion:**

Artificial intelligence-based auto-segmentation programs showed high-quality accuracy and provided standardization and efficient support for guideline-based target volume contouring as a precondition for fully automated workflows in radiotherapy treatment planning.

## Highlights

• Deep learning software show high-quality accuracy and standardization for target delineation.
• Resulting geometries required no or only minor corrections in 64–78 % of the cases.
• Clinical usability significantly correlated with the DICE (cut-off of 0.82–0.86).
• Implementation with adherence to institutional workflows seems feasible.

## 1 Introduction

The combination of manual image segmentation and target volume delineation is routinely performed for radiotherapy (RT), but a time-consuming process. Automated atlas-based techniques and artificial intelligence with deep learning tools have recently emerged, and they represent advances in radiotherapy that might offer more efficient and faster ways of planning algorithms [1], [2]. The advanced development of neuronal networks [3] and deep learning models [4] could significantly overcome the limitations of purely atlas-based programs with their simpler image segmentation algorithms and have been reported to increase efficiency and performance [5]. Current reviews provide basic knowledge and recommendations of possible validation workflows to integrate these applications into RT processes [1]. However, there is no standardized consensus for evaluating the efficiency and clinical benefit of AI-based auto-segmentation and the requirements for workflow implementation into clinical routine for specific tumor entities.

For breast cancer patients, international guidelines [6] have been published to aid radiation oncologists in achieving consistent target volume contouring in clinical routine, considering anatomical boundaries and oncological outcomes. However, studies have revealed a large and substantial variability between institutions and observers in the delineation of organs at risk (OAR) and target volumes in breast cancer RT contouring [7]. As the discrepancies have been reported to be clinically and dosimetrically significant, there is a great need for standardization.

Auto-segmentation tools have the potential to reduce variability when they provide reproducible, high-quality geometries [8]. Some studies [9] have reported an improvement for the consistencies of geometric volumes amongst different radiation oncologists with the implementation of deep learning software models.

The frequently used metric indices for quality assessment include geometric overlap or surface distance measurements [10] with the Dice similarity index (DICE), the Hausdorff distance [11], dosimetric comparisons of RT plan coverage, measurements of the time required for contouring [12] and an evaluation with a scoring system for clinical usability [8], [13], [14].

The primary aim of this study was to validate the quality and performance of three different externally trained, commercially available segmentation models for target volume contouring for the postoperative RT of breast cancer patients with regional nodal irradiation. We further aimed to show ways to enable an implementation of the tools into in-house clinical routine in order to standardize and speed up guideline-based contouring and to provide data of possible pitfalls and limitations as well as data on which metrics to use for evaluation in breast cancer patients.

## 2 Materials and methods

### 2.1 Patient population and RT treatment

Fifty consecutive patients with breast cancer who received postoperative local RT including regional nodal irradiation at our institution from 2020 to 2023 were included. Data collection and analysis were approved by the local ethics committee (S-535/2021). All patients underwent upfront tumor resection with breast-conserving surgery, simple mastectomy or mastectomy with immediate implant-based breast reconstruction and sentinel lymph node excision or axillary nodal dissection. The operative procedure and recommendation for RT were confirmed in an interdisciplinary oncological conference that included gynecologists, oncologists, pathologists, radiologists and radiation oncologists.

Radiotherapy treatment was performed with photon volumetric modulated arc therapy (VMAT) in a supine and arms-up position with an immobilization WingSTEP (IT V, Innsbruck, Austria) device. A deep inspiration breath hold technique or free breathing was used at the discretion of the radiation oncologist.

Computer tomography images were acquired with Siemens Somatom Confidence and Sensation Open (Siemens Healthineers, Erlangen, Germany) and included institutional disease-specific 120 kV scan protocols with 3 mm slice thickness with a constant tube current of 300 effective mAs using a Br40 kernel without contrast agent. Target volume delineation during clinical routine was performed by ten different well-trained board-certified radiation oncologists with each at least five years of experience and was contoured according to international European Society of Radiation Oncology (ESTRO) [6] guidelines. This included the residual breast, thoracic wall or implant after mastectomy as well as the regional lymph node areas including axilla levels 1 to 3, the supraclavicular region (level 4), the interpectoral nodes and the internal mammary node (IMN) region.

No changes were made to the original target volumes of the fifty consecutive reference cases for this analysis. An institutional peer-review process of at least two radiation oncologists is implemented during clinical routine to guarantee quality, consistency and compliance to ESTRO guideline.

### 2.2 Segmentation models and validation

Three different commercially available, artificial intelligence-, ESTRO-guideline-based segmentation models (M1-3) were applied to fifty reference CT scan cases for the delineation of clinical target volumes (CTVs):

Deep learning algorithm software of the RayStation treatment planning system (Model 1) (TPS, version 11B, RSL Breast CT 11B (v1.0.0.1), RaySearch Laboratories), local server and deep learning-based auto-segmentation software Limbus (Model 2) (Limbus Contour, Limbus Contour 1.8.0-B2, Limbus AI Inc., Regina, SK, Canada), and guideline based deep learning auto-segmentation software MVision (Model 3) (MVision AI, Version 1.2.2, Helsinki, Finland).

Seven CTVs were automatically generated for every patient: (1) the residual breast, implant or chestwall, (2) axilla level 1, (3) axilla level 2, (4) axilla level 3/infraclavicular region, (5) level 4/the supraclavicular region, (6) the interpectoral nodes, and (7) the internal mammary nodes (IMN) region.

Further, according to institutional standards, an evaluation planning target volume (ePTV) was generated and evaluated, encompassing a 5 mm margin around all seven CTVs with a skin subtraction of 3 mm for residual breast and implants and 2 mm for chestwall RT.

The created geometric structures were further evaluated via objective scoring and a manual qualitative assessment of usability. An objective validation was conducted in terms of each software model’s performance in creating geometric structures and included a comparison with the original reference volumes. For this, the DICE and the Hausdorff distance to quantify the differences and overlaps were calculated. As the DICE measures the overlap of the reference volume (X) and the automatically generated structure (Y), a value of 0 indicates no overlap, and a value of 1 defines complete overlap [8], [10], [12]. For the calculation of the DICE the following formula was used:

$$\text{DICE}\left(\text{X}|\text{Y}\right)=\text{2}\left|\text{X}\cap \text{Y}\left|/(\right|\text{X}+\text{Y}\right)$$

The Hausdorff distance [11] defines the maximum mismatch distance between two points on the surface of geometries, resulting in a perfect accuracy with a value of 0 mm, and was calculated as follows for two point sets X = {a_1_, a_2,_ …, a_NX_} and Y = {b_1_, b_2_, …, b_NX_} with a metric space d:

$$\text{Hausdorff}\;\left(\text{X},\text{Y}\right)={\text{max}}_{\text{a}\in \text{X}}\left[{\text{min}}_{\text{b}\in \text{Y}}\left\{\text{d}\left(\text{X},\text{Y}\right)\right\}\right]$$

Some studies [8] have suggested that this index is more responsive to display boundary limitations. The calculation and determination of the parameters were performed using build-in functions in RayStation accessed via the scripting interface.

All volumes resulting from model segmentations were reviewed and scored manually by one clinical radiation oncologist with at least eight years clinical experience for accuracy and usability, and scored in terms of: “no adjustments needed”, “minor corrections needed”, “major corrections needed” or “not usable”.

Moreover, influencing factors including planning-specific parameters such as deep inspiration breath hold or free breathing, patient-specific factors such as right- or left-sided tumor location, and volumetric assessments of the reference and resulting geometries were assessed and analyzed.

### 2.3 Statistical analysis

The DICE and Hausdorff metrics for all CTVs of each model were calculated with formulas and scripting interface described above. For a comparison for differences between the three auto-segmentation models and the manual reference geometries and an evaluation of the volumes of the resulting and reference geometries the Wilcoxon signed rank test was used. A p-value of less than 0.05 was considered statistically significant. To assess categorical data (right vs. left side, free breathing vs. deep Inspiration breath hold, …) the Pearson Chi-Square tests were used. Overall, computation of descriptive data was performed and box plots or tabular listing used for graphical representation of the data.

For the manual scoring of clinical usability various scoring systems have been previously published using a 3-point [15] or 4-point scale [2]. According to Almberger et al. [2] we used a 4-point scale with the following definitions:

0: “no adjustments”: deep learning volume requires no correction
1: only “minor” corrections needed
2: “major” corrections needed, but still saving time, when using as a starting point.
3: contour “not usable”

ROC (receiver operating characteristic) curve analysis was performed to calculate cut-off values for the ePTV for the DICE and Hausdorff values as predictors to geometries that were defined as “no” or “only minor corrections needed” in manual qualitative assessment of usability. For statistical calculations statistical software IBM SPSS (Armonk, NY, USA, version 28) was used.

## 3 Results

### 3.1 Study population

A total of 50 consecutive reference cases, consisting of 19 patients (38 %) with a simple mastectomy, 17 patients (34 %) with breast-conversing surgery and 14 women (28 %) with immediate implant-based breast reconstruction, were selected retrospectively and included in this study. Detailed patient characteristics and volume parameters are presented in Table 1.

**Table 1:** Patient and planning characteristics.

Characteristics	Value, percentage or range
**Postoperative status**	
chestwall	19 (38 %)
residual breast	17 (34 %)
implant	14 (28 %)

**Tumor and target localization**	
right breast	31 (62 %)
left breast	19 (38 %)

**Applied breathing technique**	
free breathing	13 (26 %)
Deep inspiration breath hold	37 (74 %)

**Reference volume**	
CTV chestwall	422 (173–833) cm^3^
CTV residual breast	675 (356–2009) cm^3^
CTV implant	968 (507–1294) cm^3^
CTV Level I	71 (35–148) cm^3^
CTV Level II	23 (5–52) cm^3^
CTV Level III	14 (4–28) cm^3^
CTV Level IV	15 (4–32) cm^3^
CTV IMN	9 (3–29) cm^3^
CTV interpectoral	13 (3–28) cm^3^
ePTV	1048 (526–2843) cm^3^

CTV: clinical target volume, IMN: internal mammary nodes, ePTV: evaluation-planning target volume. For n = 6 patients with mastectomy interpectoral nodal region were not contoured in reference CT and excluded for CTV interpectoral evaluation.

All patients received normofractionated RT and a total dose of 50.0 Gy in 25 once-daily fractions over five weeks. A simultaneously integrated boost with a total dose of 60.0 Gy was administered to two patients with suspected axillary lymph node metastases in the planning CT scans. No locoregional recurrence within the breast was present in the planning CT images after surgery.

### 3.2 Resulting geometries, DICE and Hausdorff indices

**Fig. 1:**
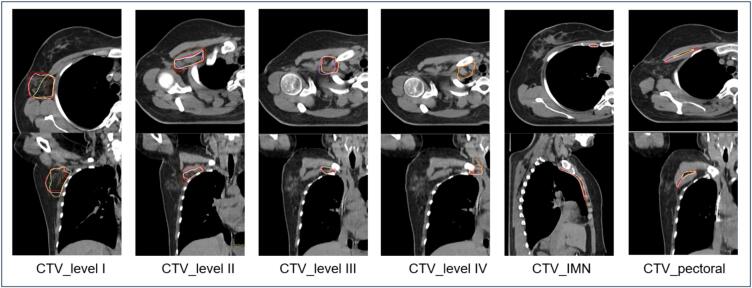
Axial and coronal CT images with resulting geometries of the CTVs for regional nodal irradiation for M1-3 (M1: pink, M2: brown, M3: yellow) and reference volume (red).

Fig. 1 displays a representative example of the resulting CTVs of the regional nodes.

Table 2 shows the volumes of the resulting geometries for each model. The size of the geometries varied significantly from the reference structures, except for the volume of CTV IMN for M1 and M3 and the volume of CTV level III for M2.

**Table 2:** Resulting volumes of all models M1-3 in cm^3^ and corresponding p-values in comparison to the reference volumes.

**Characteristic**	**Reference**	**M1**	**M2**	**M3**
CTV chestwall	422 (173–833)	157 (52–673), p < 0.001	150 (15–718), p < 0.001	229 (112–638), p < 0.001
CTV residual breast	675 (356–2009)	471 (204–1724), p < 0.001	607 (275–2139), p < 0.001	594 (277–1812), p < 0.001
CTV implant	968 (507–1294)	669 (297–942), p < 0.001	882 (401–1262), p < 0.001	783 (388–1152), p < 0.001
CTV Level I	71 (35–148)	65 (25–119), p = 0.003	83 (37–163), p = 0.001	54 (25–97), p < 0.001
CTV Level II	23 (5–52)	17 (7–34), p < 0.001	18 (4–37), p = 0.007	16 (7–27), p < 0.001
CTV Level III	14 (4–28)	13 (6–20), p = 0.012	13 (1–27), p = 0.846	15 (8–26), p = 0.003
CTV Level IV	15 (4–32)	11 (4–18), p < 0.001	19 (8–32), p < 0.001	17 (7–29), p = 0.044
CTV IMN	9 (3–29)	10 (3–12), p = 0.506	4 (2–15), p < 0.001	9 (4–12), p = 0.0**55**
CTV interpectoral	13 (3–28)	8 (2–22), p = 0.003	8 (2–32), p = 0.001	6 (1–17), p < 0.001
ePTV	1048 (526–2843)	888 (355–2371), p < 0.001	935 (117–2664), p < 0.001	873 (375–2338), p < 0.001

**Fig. 2:**
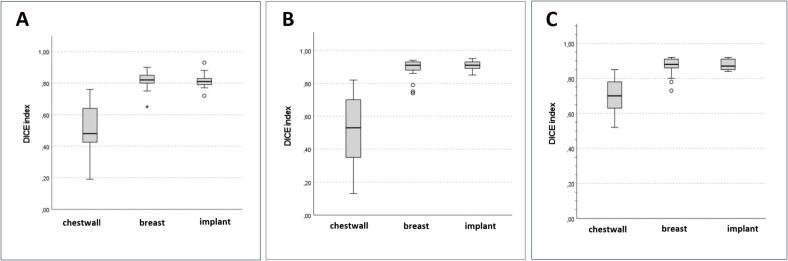
Shows boxplots with minimum, maximum and median values (line) of the calculated dice indices for the ctv of the chestwall, residual breast and implant for m1 (a), m2 (b) and m3 (c).

Table 3 illustrates the resulting DICE and Hausdorff distance values for each model in comparison to the reference cases for all CTVs and the resulting ePTV. The highest DICE values, indicating a good overlap, were found for the contouring of the residual breast and implant for all models (M1: 0.82 and 0.81; M2: 0.91 and 0.91; M3: 0.88 and 0.87). Of the analyzed structures, the lowest DICE and highest Hausdorff distances were found for the CTV chestwall, representing significantly strong deviations from the reference structure for all models (Fig. 2).

**Table 3:** DICE index und Hausdorff distance.

**Characteristic**	**Model 1**		**Model 2**		**Model 3**	
	**DICE** **median (range)**	**Hausdorff** **median (range)**	**DICE** **median (range)**	**Hausdorff** **median (range)**	**DICE** **median (range)**	**Hausdorff** **median (range)**
CTV chestwall	0.48 (0.19–0.76)	4.99 (3.29–9.41)	0.53 (0.13–0.82)	7.31 (2.33–13.08)	0.70 (0.52–0.85)	3.69 (2.42–9.63)
CTV residual breast	0.82 (0.65–0.90)	3.33 (2.14–4.56)	0.91 (0.74–0.94)	2.63 (1.51–6.15)	0.88 (0.73–0.92)	3.14 (1.98–4.37)
CTV implant	0.81 (0.72–0.93)	3.39 (1.43–5.01)	0.91 (0.85–0.95)	2.71 (1.94–4.94)	0.87 (0.84–0.92)	2.82 (2.17–4.23)
CTV Level I	0.74 (0.54–0.87)	2.18 (1.09–4.52)	0.75 (0.55–0.84)	2.14 (1.33–4.52)	0.68 (0.48–0.77)	2.17 (1.15–3.71)
CTV Level II	0.67 (0.27–0.79)	1.76 (0.76–5.80)	0.62 (0.25–0.76)	2.09 (1.00–5.22)	0.63 (0.32–0.78)	1.86 (1.00–5.01)
CTV Level III	0.68 (0.29–0.83)	1.50 (0.64–4.27)	0.59 (0.11–0.76)	3.23 (1.08–7.05)	0.71 (0.26–0.89)	1.45 (0.70–4.51)
CTV Level IV	0.59 (0.15–0.86)	1.70 (0.92–2.97)	0.57 (0.27–0.78)	1.68 (0.90–3.19)	0.64 (0.33–0.77)	1.66 (0.84–3.41)
CTV IMN	0.64 (0.40–0.76)	1.88 (0.65–3.54)	0.37 (0.19–0.60)	2.32 (0.76–5.75)	0.61 (0.41–0.72)	2.90 (0.76–7.96)
CTV interpectoral	0.62 (0.31–0.80)	2.46 (0.96–6.42)	0.51 (0.22–0.78)	2.36 (0.59–5.63)	0.51 (0.06–0.73)	2.54 (1.22–5.92)
ePTV	0.88 (0.60–0.93)	3.20 (1.71–9.24)	0.87 (0.52–0.99)	3.08 (1.91–23.63)	0.87 (0.66–0.93)	2.96 (1.94–9.80)

CTV: clinical target volume. DICE: Dice similarity index, IMN: internal mammary nodes. ePTV: evaluation-planning target volume.

The nodal irradiation volumes with the lowest DICE values were as follows: M1: CTV level IV at 0.59; for M2 CTV IMN at 0.37; and CTV interpectoral for M3 at 0.51. This did not reflect the results of the corresponding nodal CTVs with the highest Hausdorff distances for M1 CTV interpectoral at 2.46; for M2 CTV level III at 3.23, and for M3 CTV IMN at 2.90.

The median DICE and Hausdorff metrics for the resulting ePTV were comparable for all models: M1 0.88 (range: 0.6–0.93), for M2 0.85 (range: 0.38–0.92) and for M3 0.87 (range: 0.66–0.93) for the DICE. For M1 3.20 (range: 1.71–9.24), for M2 3.08 (range: 1.91–23.63) and for M3: 2.96 (range: 1.94–9.80) for the Hausdorff distance.

### 3.3 Clinical evaluation

A manual evaluation of the clinical usability of all model CTVs was conducted, and the scores are listed in Table 4. Clinical use after only minor or no adjustments was possible in 72 % (M1), 64 % (M2) and 78 % (M3) of the cases, respectively. Only in four cases were the contours defined as not usable with model M2, including two cases where no structure of the chestwall was created. The most detected deviations were in the two most cranial and caudal layers of each CTV volume of all models.

**Table 4:** Scoring of clinical evaluation for usability and cut-off values.

**Corrections**	**Model 1**	**Model 2**	**Model 3**
no	7 (14 %)	6 (12 %)	12 (24 %)
minor	29 (58 %)	26 (52 %)	27 (54 %)
major	14 (28 %)	14 (28 %)	11 (22 %)
not usable	0 (0 %)	4 (8 %)	0 (0 %)
**Sum: no/minor**	72 %	64 %	78 %

**Cut-off value for no/minor**			
DICE	0.86	0.85	0.82
Hausdorff	3.04	3.97	3.30

The manual assessment of the clinical usability performed by the radiation oncologist correlated significantly (p < 0.001 each) with the DICE and Hausdorff scores for the ePTV for all models (DICE: M1: p < 0.001, M2 and M3: p < 0.0001. Hausdorff: M2 and M3: p < 0.0001) except for the Hausdorff index for M1 (p = 0.117).

Cut-off values for the DICE and Hausdorff values were calculated for the ePTV to define the need to make no or only minor corrections and presented in Table 4: M1 had a DICE of 0.86 and a Hausdorff of 3.04; M2 had a DICE of 0.85 and a Hausdorff 3.97; and M3 had a DICE of 0.82 and a Hausdorff of 3.30.

### 3.4 Influencing factors

There were no significant differences in the objectively and manually scored quality of the contouring of right- or left-sided volumes or in the applied RT breathing technique (DIBH or FB) for all three models on the DICE and Hausdorff indices. As an example, the DICE values of the CTV for the residual breast were comparable with 0.86 (M1), 0.93 (M2) and 0.91 (M3) for the right side and 0.82 (M1), 0.90 (M2) and 0.87 (M3) for the left side, respectively.

Of note, oncological abnormalities in the reference planning CT were described in 10 patients, requiring minor deviations from the ESTRO guidelines, with an enlargement of contouring volumes in 4 of them for the expansion of the target area due to a preoperative very caudal tumor site (n = 1), suspicious enlarged lymph nodes at the supraclavicular level (n = 1), an axillary residual lymph node tumor (n = 1) and suspicion of deep pectoralis muscle infiltration (n = 1).

## 4 Discussion

While artificial intelligence already aids in cancer detection in imaging, automated tools and segmentation models offer further enormous advantages in radiation oncology in terms of increasing efficiency and standardizing target volume contouring.

Our study focused on the accuracy of the resulting structures and implementation in internal workflow standards, and it presented all three commercially available segmentation approaches to maintain high-quality guideline-based contouring for the postoperative RT of patients with breast and regional nodal irradiation with no or only minor corrections needed in approximately two-thirds of them. Only the automatic contouring of the chest wall after simple mastectomy could not produce clinically satisfactory results.

Objective and manual evaluations of the models confirmed that they created reliable geometries with adherence to local standards and institutional workflows, indicating that their implementation into daily practice is easily feasible.

Our results are approximately in line with the sparse previously published data. Almberg et al. [2] validated a cohort of 30 patient cases with a deep-learning segmentation model for loco-regional breast cancer and found that 14 % required no adjustments and 71 % only minor corrections of the CTVs. They reported that 15 % required major modifications, while our study revealed the need for more time-intense re-contouring in 22–28 % of the patients. However, the cranial and caudal aspects of the geometries were confirmed as needing the most frequent adjustments in our study.

The automated delineation of OARs including the lungs, heart and breast, has already been well established in radiotherapy. A study by Radici et al. [16] reported the implementation and effects of automated OAR delineation with Limbus software in head and neck, prostate, left-side breast and rectal cancer. The largest time saving was achieved in patients with head and neck tumors, with a 65 %-time reduction, while the largest dosimetric difference was observed in patients with rectal cancer. Regarding the time required for OAR contouring in breast cancer patients, the authors found a 46 % reduction.

Strolin et al. [9] assessed 20 breast cancer patients with MVision deep learning software delineated targets compared to manual contouring and found a median and maximum percentage of time save of 48 % and 63 %, respectively, with a −8.7 min time difference for auto-segmentation including subsequent manual corrections.

Comparable results were also shown by Bakx et al. [15], who were able to achieve an overall time reduction of 92 % with the use of deep learning auto-segmentation RaySearch models for breast cancer for the CTV including lymph node regions (42 % time reduction, absolute −8 min) and OAR (58 % time reduction, absolute −25 min). Even though the models were highly beneficial for RT workflows and clinical routine, the absolute time required for manual contouring highly depends on the experience of the radiation oncologist.

Target volume contouring in tumors of the head and neck has also been reported [17] to be highly individual depending on the radiation oncologist's experience and standardization is highly needed, as studies have confirmed its impact on oncological outcome, with improved overall survival in experienced centers. Auto-segmentation in head and neck tumor contouring resulted in significantly smaller interobserver delineation differences and dosimetric variations [18].

These results indicate that auto-segmentation-based contouring might be able to improve RT treatment quality and oncological outcome. However, the atlas- and deep learning-based models used and available differ significantly. Choi et al. [5] compared the efficacy and accuracy of atlas-based versus deep learning-based models in women with breast cancer for the contouring of target volumes and organs at risk. In their analysis, the deep learning model yielded more consistent and robust results, especially for the CTVs and the substructure of the heart. Liu et al. [3] trained an in-house convolutional neuronal network to automatically delineate the chestwall CTV with 110 CT scans in female postmastectomy patients and did a blind evaluation of the models output in 10 randomly selected patients. With a contouring time of only 3.45 s, they concluded that the AI-generated structures were comparable or even better than the manual volumes.

Overall, commercially available products are mostly trained on external data sets outside the institution to which they are to be integrated. Another study of Bakx et al. [19] compared the difference of manual delineations of organs at risk and target volumes for 30 breast cancer patients to an externally trained model and to an in-house trained model. Although the results revealed statistically significant variances between the two models, these were shown to be within an acceptable range within inter-observer variations and the authors proved clinical usefulness for both models.

However, the quality and complexity of automated contouring vary depending on the region to be irradiated and the type of tumor, and individualized studies are required for each tumor entity and software model. Our median DICE and Hausdorff values for the ePTV were 0.87– 0.88 and 2.96–3.55, with cut-off DICE values of >0.8, which we considered of high clinical acceptability in our institution. A further assessment of how this is reflected in dosimetric deviations is currently being analyzed. The aforementioned study of Bakx et al. [15] reported a DICE of 0.92 (±0.06) for the right-sided target breast CTV with deep learning auto-segmentation RaySearch models and scored the results as clinically acceptable in 92 %. They further analyzed the effect of additional manual correction of the resulting auto-segmented structures and proved only low improvement to the DICE of only 0.02 (±0.05). The previously mentioned study of Liu et al. [3] reported a DICE for the chestwall of 0.9 and a Hausdorff value of 5.65 mm. Two experienced radiation oncologists evaluated the AI-generated CTV slices with different acceptance rates of 97.9 % (no revision) and 1.4 % (minor revision) and 89.1 % (no revision) and 9.8 % (minor revision), respectively. Further, Chung et al. [20] assessed deep learning auto-segmentation for 111 women with breast cancer and rated a mean DICE of 0.8 for OAR and a mean DICE for all breast and regional lymph node CTVs of higher than 0.7 as clinically acceptable.

Our study confirmed that the various objective metrics were not consistently correlated with each other or with subjective evaluations and used inconsistently in the literature. For individual cases and challenges of different tumor extensions or specific treatment regions, volumetric and distance measurements might not be well correlated with each other, so statistical validation for clinical implementation and acceptability should not only be based on one single index [8], is highly subjective and there is a need for more consensus of auto-contouring implementation and evaluation [14].

In this context, more critical reviews [21] oppose a strict threshold value of geometric indices for clinical acceptability, while other studies have proposed a clinically acceptable cut-off value for usability for the DICE also in other tumor entities of approximately 0.7 for cervical [22] and pancreatic cancer [23].

Moreover, assessments of an internal validation cohort in multiple domains including institutional-specific objective geometric evaluations and multidisciplinary reviews by radiation oncologists, physicians and physicists are required to guarantee a safe and clinically relevant application and implementation.

The limitations of this study include the heterogeneity and potential intrinsic bias of the reference cases that were contoured during clinical routine by different radiation oncologists and the subjective manual evaluation of the usability potentially influenced by the radiation oncologist’s experience or in-house workflows. Moreover, the use of scoring systems for clinical acceptance and the assessment of usability is highly subjective and biased by inter-observer variations. However, regardless of international contouring guidelines, the implementation of auto-segmentation tools has different requirements and institutional standards depending on the individual department, which are best reflected by reviewing internal reference cases to objectively find the model that fits best to the internal workflow.

Our study further presents the results of the delineation of the shape of the residual chestwall after simple mastectomy, which has not yet been officially included in the current commercially available contouring programs and which, according to our study, cannot be replaced by the provided breast volume geometry tool.

Since this study was performed retrospectively, a systematic measurement of the exact time reduction between manual and automatic segmentation for each patient was not feasible. However, manual target volume delineation in clinical routine highly depends on the experience of the treating radiation oncologist as well as possible distracting or unforeseen events that can often occur during clinical routine. The time needed to generate the auto-segmented structures was in the range of minutes within the used local server or upload platform in each model and comparable for M1-3. Of note, different informatic hardware and software requirements for CT scan exports or imports into routine planning systems in different institutions might be challenging or more time-consuming.

The combined development and improvement of automated target volume contouring is an approach and precondition that contributes to the aim of performing fully automated strategies in RT treatment planning. While this enables improvements in imaging processing and automated segmentation and planning workflows, implications for practice include the need for a critical and thorough evaluation of the resulting output by the treating radiation oncologist and a review for necessary deviations from guidelines, as well as the challenge of constantly needing to update and adapt to changes in guidelines or research findings. Auto-contouring solutions for repetitive tasks seem highly attractive for workflow implementation and advantageous for preventing human errors, the restructuring of staff resources, enhanced and efficient productivity, and the standardization of and a reduction in the contouring and planning time.

Clearly, there are a number of challenges with auto-segmentation solutions that include IT requirements for hardware and software, the necessity to constantly update and adapt to changes in guidelines or research findings. Furthermore, complex patient cases may sometimes require more elaborate workups and clinical decisions that deviate from the norm or guidelines.

## 5 Conclusion

In this study, artificial intelligence-based auto-segmentation programs were shown to have high-quality accuracy and provided helpful standardized and efficient support for guideline-based target volume contouring in breast cancer patients with regional nodal irradiation. Implementation into clinical workflow seemed easily feasible with the maintenance of both internal institutional standards and international guidelines. Our study provides clinical validation as a precondition for fully automated workflows in radiotherapy treatment planning. However, thorough clinical, institutional-specific evaluations, visual inspection and manual corrections of the resulting output as well as possible required deviations due to oncological abnormalities by the treating radiation oncologist and the constantly needed updates remain important issues. Further, analyses to what extent the deviations of automatically generated contours translate into clinically relevant dose distribution differences or effects on oncological outcome are needed.

## Declaration of competing interest

The authors declare the following financial interests/personal relationships which may be considered as potential competing interests: Our radiotherapy department has on-going research collaboration with RaySearch Laboratories AB. All authors affirm that they have no financial, non-financial or personal interest or belief in the subject matter or materials discussed in this manuscript, that might jeopardize their objectivity. EM received speaker fees from Elekta outside the submitted work. SK received speaker fees from Siemens Healthineers. JL received travelling support from Micropos Medical and from RaySearch Laboratories outside the submitted work. FW received speaker fees from AstraZeneca, Varian Medical Systems and Merck Sharp & Dohme and travel support for attending meetings from Varian Medical Systems and Micropos Medical outside the submitted work. PHS received support from Physician Scientist Program of the Medical Faculty (University of Heidelberg), grants from Dietmar-Hopp-Foundation and fees from NovoCure GmbH outside the submitted work. JD reports grants from CRI The Clinical Research Institute, grants from View Ray Incl., grants from Accuray International, grants from Accuray Incorporated, grants from RaySearch Laboratories AB, grants from Vision RT limited, grants from Merck Serono GmbH, grants from Astellas Pharma GmbH, grants from Astra Zeneca GmbH, grants from Siemens Healthcare GmbH, grants from Solution Akademie GmbH, grants from Eromed PLC Surrey Research Park, grants from Quintiles GmbH, grants from Pharmaceutical Research Associates GmbH, grants from Boehringer Ingelheim Pharma GmbH Co, grants from PTW-Frieburg Dr. Pychlau GmbH, grants from Nanobiotix A.a., grants from IntraOP Medical, outside the submitted work. LK reports, personal fees from Accuray Inc., and Novocure GmbH outside the submitted work. JHR reports honoraria and travel reimbursement by Viewray Inc., Pfizer Inc and IntraOP Medical as well as grants from IntraOP Medical and Varian Medical Systems outside the submitted work. BG, FRW, DN, LH, LAD, SH, MV have nothing to declare.
